# Usability evaluation of the educational website “understanding my diabetes” for Mexican patients with type 2 diabetes

**DOI:** 10.3389/fpubh.2024.1394066

**Published:** 2024-05-10

**Authors:** Gabriela Ortíz Ortíz, Sandra Vega García, Cristina Islas Salinas, Abril Violeta Muñoz Torres, Lubia Velázquez López

**Affiliations:** ^1^Unidad de Investigación en Epidemiología Clínica, Hospital General Regional No. 1 “Dr. Carlos Mac Gregor Sánchez Navarro” Instituto Mexicano del Seguro Social, Ciudad de México, Mexico; ^2^Unidad de Medicina Familiar Número 7, Coordinación de Educación e Investigación en Salud, Instituto Mexicano del Seguro Social, Ciudad de México, Mexico; ^3^Departamento de Salud Publica, Facultad de Medicina, Universidad Nacional Autónoma de México, Ciudad de México, Mexico

**Keywords:** type 2 diabetes, health information technology, usability, website, diabetes education

## Abstract

**Background:**

Diabetes education is an integral part of the treatment for the metabolic control of patients with diabetes. The use of the Internet as a tool for diabetes education, as well as its acceptance, is still under study.

**Aim:**

To assess the usability of the educational website “I understand my diabetes” designed for patients with type 2 diabetes attending primary care clinics.

**Material and method:**

A cross-sectional study was done in 110 patients with type 2 diabetes from two family medicine clinics, each of whom was assigned a user account on the educational website “Entiendo mi diabetes.” The web site assigned a user name and password to each patient. They were able to access the educational website at home. After a 15-day review period, participants were asked to evaluate usability using the Computer System Usability Questionnaire. Additionally, we developed an eight-item questionnaire usability focusing on diabetes care. Sociodemographic data, blood pressure, and anthropometric measurements were recorded. Glucose levels and lipid profiles were also measured.

**Results:**

The patients with diabetes had a mean age of 52.7 years and a median of 5 years since they were diagnosed with diabetes. The website received a good usability rating from 89.1% of participants, with favorable assessments in all three dimensions: 87.3% for information, 85.5% for quality, and 88.2% for interface. Regarding usability specifically for diabetes care, 98.2% rated it as having good usability.

**Conclusion:**

The website for education about the disease in patients “I understand my diabetes” had an adequate usability evaluation by patients, so they also considered it very useful for diabetes care. The diabetes care instrument had adequate usability and reliability.

## Introduction

Type 2 diabetes (T2D) is defined as a group of metabolic disorders characterized by chronic hyperglycemia. It is the result of impaired insulin secretion, deficient effectiveness, or both conditions (1). Globally, in 2019, its estimated prevalence was 9.3%, affecting 463 million people, and it is projected to increase to 700 million by 2045. Moreover, it is estimated that approximately 50.1% of individuals with T2D are unaware of their condition (2).

In Mexico, the prevalence of T2D was 9.5% in 2016, and 18.3% (5.8% were new diagnosis) in 2022 (3). The objectives of comprehensive treatment are to achieve glycemic control with a glycated hemoglobin (HbA1c) level < 7%, with a primary focus on reducing long-term micro and macrovascular complications (4).

In the 2016 National Health and Nutrition Survey (Ensanut 2016) the researchers reported that 68.2% of the population diagnosed with diabetes in Mexico has poor metabolic control (HbA1c ≥ 7%) (5). Since this persistent poor glycemic control, the increase in the prevalence of T2D and the late diagnosis of the disease, it is necessary to promote strategies to adopting a healthy lifestyle and disease care. Metabolic control in patients with diabetes must have a comprehensive approach, according to the physician care, pharmacological treatment, and initiatives to promote a healthy lifestyle.

Diabetes education is a crucial component for instilling knowledge about the disease, promoting self-care, and encouraging positive lifestyle changes (6, 7). Furthermore, evidence suggests that providing diabetes education reduces the risk of condition related mortality (8).

Over the past two decades, there has been a growing use of Health Information and Communication Technologies (ICTs) for providing diabetes education and monitoring indicators (9). It has been demonstrated that these tools can contribute to improving HbA1c levels, body composition, knowledge of the disease, and the adoption of a healthy lifestyle (9, 10).

Nevertheless, it has been reported that educational websites lack validated information, have accessibility issues, and exhibit variability with regard to the quality of the information, its updating, ease of use and comprehension (11, 12).

The term “usability” is defined as the degree to which users can perform tasks accurately and efficiently (13). According to the International Organization for Standardization (ISO 9241), usability refers to the efficiency, effectiveness, and satisfaction of users in a specific context (14). Usability reports exist for developments involving the use of ICTs in diabetes, indicating a range from 38 to 80% (15, 16). Limitations include difficulty completing multiple steps to achieve an objective, limited functionality and interaction, navigation challenges, as well as a high abandonment rate. In addition, patient difficulties in accessing and experiencing educational websites have been documented (17).

The persistent increase of patients with diabetes makes it difficult to provide education about the disease in primary care; therefore, implementing strategies with the use of ICTs, designed by clinicians with experience in diabetes, as well as in the culture, educational level and social environment of the patients, becomes an imperative need. In this sense, it is required to have digital tools adapted to the end user, which are easy to use and understand, as well as useful to improve the knowledge of T2D and promote its care.

In Mexico, the usability of systems designed to provide diabetes education is even more limited than that reported in other countries. In this sense, our research group developed an educational site called “Understanding my diabetes” which was built by physicians, nutritionists, and diabetes educators with more than 10 years of experience. The diabetes web site was validated by a consensus of experts (18).

Therefore, the aim of this study is to measure the usability of the educational website “Understanding my diabetes” for ease awareness of the condition and improve self-management in patients with T2D, attending primary clinics care.

## Materials and methods

### Study design and population

A descriptive cross-sectional study was conducted among patients affiliated with two family medicine clinics of the Instituto Mexicano del Seguro Social (Mexican Institute of Social Security, IMSS) in Mexico City from September 2021 to May 2022.

The study was approved by the Ethics and Research Committee at IMSS with registration number R-2021-3609-025. Patients were invited to participate in the study during their clinic appointments. The participating researchers provided information about the study to the patients, answered their questions and, once they decided to participate voluntarily, their consent was requested by signing the informed consent form.

The sample size was calculated based on the assessment of the usability of the educational program and findings reported in a similar study (19). A formula was used for proportion, with a confidence level of 95%, an expected proportion of 1.21, and a margin of error of 0.10, resulting in a total of 92 participants. To account for a 20% potential loss (participants cannot completing the assessment), 110 patients were included in the study.

### Selection criteria

The study included patients who were previously diagnosed with T2D by their treating physician, aged 18 or older, and who had been diagnosed less than 10 years ago. Participants needed to be literate, to have access to a home computer or smartphone, and possess internet connectivity. Patients with severe complications of the disease, such as chronic kidney disease requiring replacement therapy, blindness, amputation, or any condition that hindered the evaluation of the educational website (visual impairment, or psychological conditions), were excluded.

**Figure 1:**
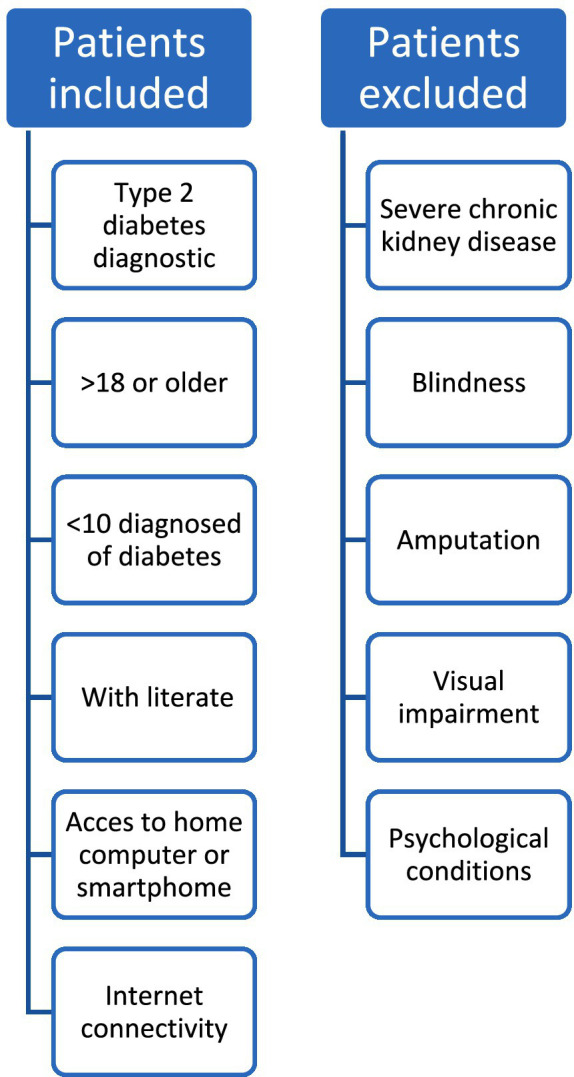
Selection criteria.

Patients with severe complications of the disease, such as chronic kidney disease (patients that requiring replacement therapy), blindness, amputation, or any condition that hindered the evaluation of the educational website (visual impairment, or psychological conditions) were excluded (Figure 1).

### Sociodemographic and clinical measurements

The collection of sociodemographic and clinical data was conducted by participating medical and nutrition staff. Blood pressure readings were recorded, measured by a healthcare professional on two occasions using a mercury sphygmomanometer, with a five-minute interval between each reading. Patients were requested to remain seated for more than 5 min before the readings. The recorded blood pressure value was the average of the two measurements.

### Socioeconomic status

The socioeconomic status was assessed using the INEGI questionnaire known as AMAI, comprising 7 levels. To categorize participants into three socioeconomic classes, a scoring system defined by the authors of the instrument was employed: a score between 0 and 111 points indicated a low socioeconomic level, 112–204 points indicated a medium level, and a score of ≥205 indicated a high socioeconomic level (20).

### Anthropometric measurements

Anthropometric measurements were recorded by two nutritionists who received prior training and standardization through a training course.

Two nutritionists took a training course for the standardization of measurements. They recorded the anthropometric measurements of the sample. They followed the Habitch method, adhering to the specifications recommended by Lohman and colleagues (21, 22). Body weight was measured on a calibrated SECA^®^ model 813 scale, with participants wearing light clothing. Height was measured using a SECA^®^ model 220 stadiometer, with the patient positioned with their back to the scale following the Frankfort plane. The body mass index (BMI) was calculated.

Waist circumference (WC) was measured with a SECA® measuring tape, with the reference point at the midpoint between the lowest rib and the upper edge of the iliac crest on the right side. Both measurements were assessed at three different times, and the mean value of the second and third measurements was used for recording and analysis.

### Biochemical measurements

Biochemical indicators were measured in venous blood after a 10-h fasting period. High-Performance Liquid Chromatography (HPLC) was employed. Automated photometry (Roche Cobas 800 c701) was used for the measurement of glucose, total cholesterol levels, and triglycerides.

Educational Website “Entiendo mi diabetes” in Spanish language. Este sitio web educativo fue desarrollado por el equipo investigador, compuesto de medicos especialistas de familia, de medicina interna, nutriólogos, epidemiologos, educadores en diabetes, informáticos, que participan con el equipo de investigación. The educational course was implemented on the Moodle platform at the website www.entiendomidiabetes.com by a team of web developers. The course comprises modules on understanding diabetes, pharmacological treatment, control indicators, nutrition, myths surrounding diabetes, complications related to the disease, depression, and family support. These modules are designed in a straightforward manner, incorporating visual resources and minimal text. The use of videos facilitates easy comprehension. Each module includes didactic or reinforcing activities with the aim of motivating patients to actively manage their health.

These educational modules were validated through an expert consensus, including physicians, nutritionists, psychologists, and diabetes educators. The validation process was conducted blindly and in pairs, assessing the content validity of the educational modules, which has been previously reported (18).

This course is managed over Moodle, the free open- source Learning Platform and can be visited at: http://entiendomidiabetes.com/moodle28/login/index.php.

### Usability measurement instrument

The level of usability was assessed using the Computer System Usability Questionnaire (CSUQ), validated in the Mexican population (23).

The instrument comprises 16 items that evaluate three main domains: system quality (items 1–6), information quality (items 7–12), and interface quality (items 13–16). The instrument utilizes a 7-level Likert scale, ranging from completely disagree “1“to completely agree “7.” In order to assess usability, a total of 112 points were used, categorized into tercile: low usability (<37 points), moderate (38–74 points), and high usability (>75 points). Regarding the dimensions, for a maximum quality score of 42 points, (low quality ≤14 points, moderate 15 to 28 points, and high quality ≥29 points). Patients created their username and password for the educational site, and after interacting with the site, from login to the complete review of one of the modules, they were asked to complete the usability questionnaire.

### Diabetes care instrument

A research team consisting of two-family physicians, two nutritionists, and two diabetes educators designed a 10-item questionnaire aimed at measuring usability for diabetes care. Following a second content analysis review, two items were eliminated as they were deemed repetitive. Eight items were included, focusing on measuring understanding of content (items 1 and 2), items directed toward positive changes in disease care (items 3–7), and likelihood to recommend the educational site to others (item 8). A Likert scale ranging from 1 to 7 was used. A score of 8–24 was considered low usability in diabetes care, 25–40 as moderate, and ≥41 as high usability.

### Statistical analysis

The data were analyzed using the statistical package SPSS, version 25. Qualitative variables characterizing the population and the type of usability of the educational program are presented in frequencies and proportions. The Kolmogorov–Smirnov normality test was employed to identify the distribution of quantitative variables. For variables with a parametric distribution, mean and standard deviation were used, while for those with a non-parametric distribution, median and interquartile range were utilized.

The usability of each item in the instrument was measured through the mean and standard deviation.

To assess the scale reliability of the diabetes usability instrument CSUQ and the instrument of diabetes care, Cronbach’s alpha was calculated.

To identify the association between usability and qualitative variables with sociodemographic and clinical variables, the chi-square test was employed. A statistically significant difference was considered with a *p*-value < 0.05.

## Results

The sociodemographic characteristics and data of the studied population are presented in Table 1.

**Table 1:** Sociodemographic characteristics of the studied population with diabetes.

	*n* (%)
**Gender**	
Females	73 (66)
Males	37 (34)
**Education level**	
Basic	31 (28)
Middle	36 (33)
High	43 (39)
**Socioeconomic status**	
Upper class	19 (17)
Middle class	74 (67)
Lower class	17 (16)
Diabetes education	14 (13)
Nutritional therapy	32 (29)
Physical activity	14 (13)
Hypertension	35 (32)
Dyslipidemia	42 (38)
Smoking	20 (18)
Alcohol consumption	19 (17)
	**Mean and standard deviation**
Age	52.7 ± 10.26
**Weight Kg**	
Females	77.5 ± 13.16
Males	81.04 ± 12.5
Cholesterol mg/dL	179.87 ± 34.9
	**Median and interquartile range**
Years since diabetes diagnosis	5 (2–8)
BMI Kg/m^2^	29 (28–32)
Glucose mg/dL	145 (120–173)
Triglycerides mg/dL	152 (126–210)

*n* = 110.

The data are presented as frequency and percentage.

BMI, Body Mass Index.

A total of 110 patients were included, 66% were female. The average age of the study population was 52.7 ± 10.2 years, with a median diabetes diagnosis duration of 5 years. The most frequently reported level of education was higher education (39%), while the most frequently identified socioeconomic level was middle class, (67%). Thirteen percent received diabetic education and 32% of the patients studied received nutritional therapy previously.

Thirteen percent received diabetic education and 32% of the sample received nutritional therapy previously. The median BMI was 29 kg/m^2^, and fasting glucose was 145 mg/dL.

Table 2 displays the rating for each item in the usability instrument of the educational site. The highest average rating was 6.47 for the item addressing assistance in completing tasks, whereas the lowest was 6.03, pertaining to resolving issues on the website. The mean of usability of the educational website was 94.76 ± 15.48 and the reliability test was 0.97 (Table 3). The usability of the website using the diabetes instrument shows that 98.2% of patients rated it as high and 1.2% as regular.

**Table 2:** Usability rating of the educational website “Entiendo mi diabetes.”

	Mean and standard deviation
Overall, I am satisfied with how easy it is to use the website	6.19 ± 1.12
This website was easy to use	6.18 ± 1.27
I am able to complete my work quickly using this website	6.21 ± 1.21
I feel comfortable using this website	6.30 ± 1.07
It was easy to learn how to use this website	6.15 ± 1.32
I think I became an expert quickly using this website	6.08 ± 1.37
The website displays error messages that clearly tell me how to solve problems	6.17 ± 1.42
Every time I make a mistake using the website, I resolve it easily and quickly	6.03 ± 1.50
The information (such as online help, on-screen messages, and other documentation) provided by this website is clear	6.33 ± 1.24
It is easy to find the information I need on the website	6.29 ± 1.25
The information provided by the website was effective in helping me complete tasks	6.47 ± 1.00
The organization of the information on the website screen was clear	6.45 ± 0.95
The website interface was pleasant	6.33 ± 1.14
I enjoyed using the website	6.41 ± 1.13
The website had all the tools I expected it to have	6.35 ± 1.18
Overall, I was satisfied with the website	6.40 ± 1.10
Mean usability	94.76 ± 15.48
Cronbach alpha	0.97

The rating for each item ranges from a minimum of 1 to a maximum of 7.

**Table 3:** Usability level of the educational site related to aspects of diabetes care.

	Mean and standard deviation
Using this website helped me understand the behavior of my diabetes	6.79 ± 0.48
Using this website helped me learn new information about my condition	6.74 ± 0.70
Using this website helped me stay motivated to improve my diabetes self-care	6.76 ± 0.58
Using this website helped me follow my doctor’s recommendations	6.74 ± 0.58
Using this website helped me take care of my body and pay attention to my condition	6.77 ± 0.66
Using this website helped me improve my diet	6.73 ± 0.74
Using this website helped me stay motivated to work-out	6.70 ± 0.77
I would recommend the use of this educational website on diabetes to my family and friends	6.71 ± 1.01
Mean usability	54.37 ± 3.94
Cronbach alpha	0.93

The rating for each item ranges from a minimum of 1 to a maximum of 7.

**Figure 2:**
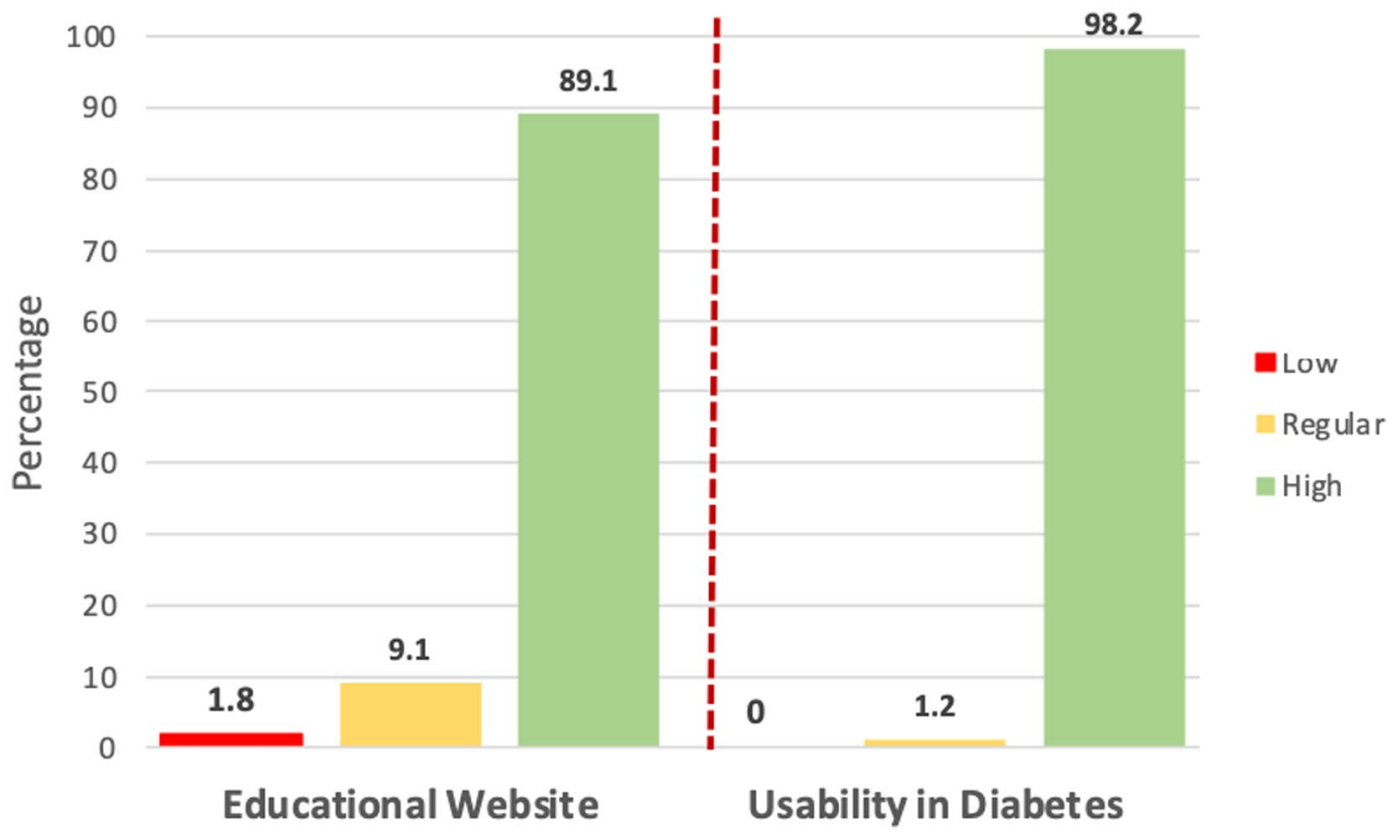
Usability level of the educational website “Entiendo mi diabetes”.

**Figure 3:**
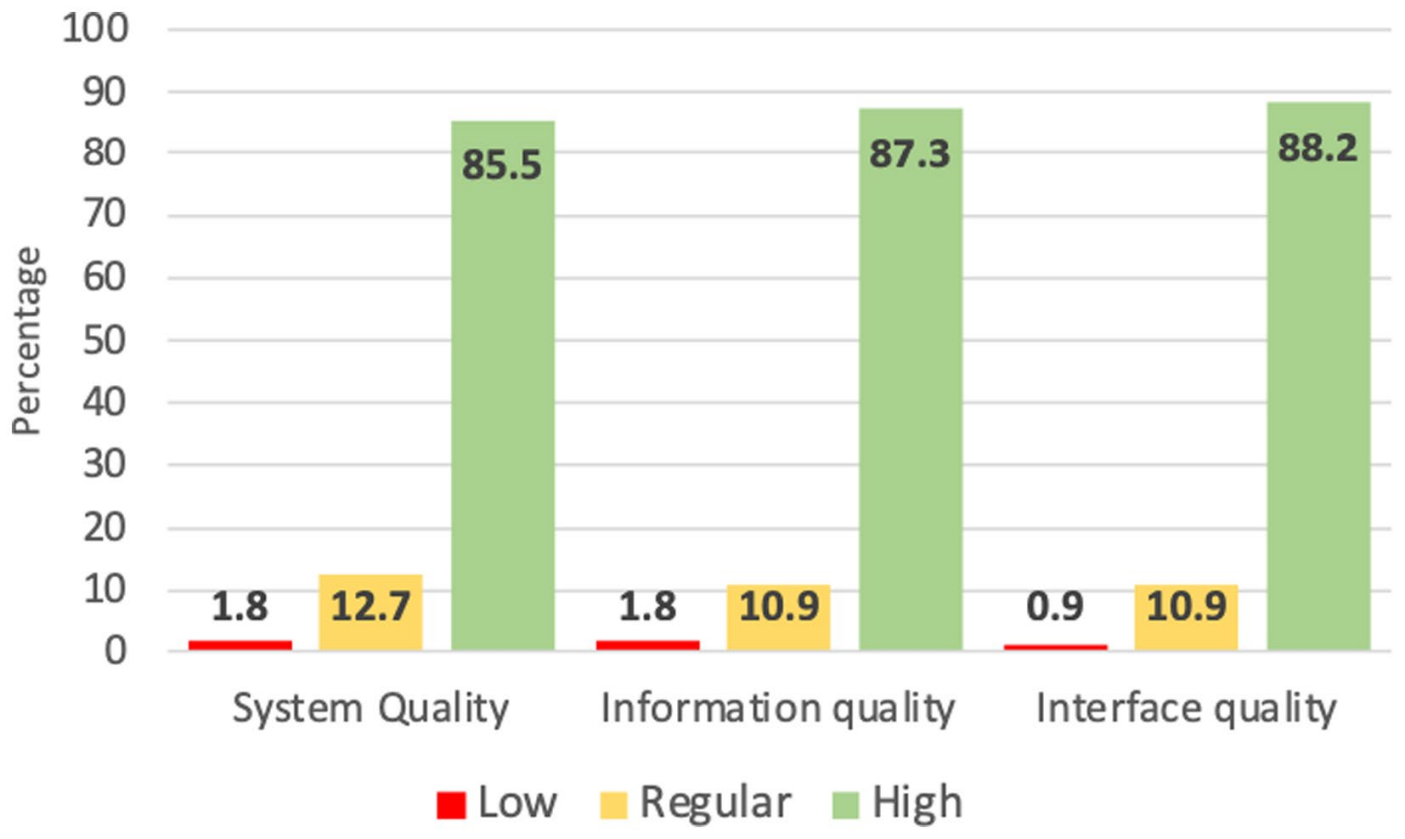
Usability in the dimensions of the website “Entiendo mi diabetes”.

The usability description is presented in Figure 2, revealing that 89.1% of the patients rated it as high usability, 9.1% as moderate, and 1.8% as low usability. In Figure 3, the three dimensions of usability from the instrument are illustrated, indicating high usability across all three dimensions: 87.3% for information, 85.5% for quality, and 88.2% for the interface.

Regarding the average rating of the 8 items in the instrument aimed at measuring usability in diabetes care, 98.2% rated it as having high usability. The highest average rating was related to understanding the behavior of diabetes (6.79), while the lowest was motivation for physical exercise (6.70). The overall mean usability of the diabetes care instrument was 54.37 ± 3.94 and the reliability value was 0.93.

Low, moderate, and high usability were compared with sociodemographic variables, prior diabetes education, socioeconomic level, education level, and marital status, with no statistically significant differences found among them. There was no difference between glycemic control and type of usability; the data are presented in Table 4.

**Table 4:** Usability level and its association with sociodemographic and clinical variables.

	Usability			
	Low	Regular	High	*p*-value
Gender				0.571
Females	1 (1.4)	8 (11)	64 (87.6)	
Males	1 (2.7)	2 (5.4)	34 (91.9)	
Diabetes diagnosis				0.215
<5 years	0 (0)	3 (5.9)	48 (94.1)	
>5 years	2 (3.4)	7 (11.9)	50 (84.7)	
Age (years)				
18–30	0 (0)	0 (0)	2 (100)	0.281
31–50	2 (4.5)	2 (4.5)	40 (91)	
>51	0 (0)	8 (12.5)	56 (87.5)	
Diabetes education				
Yes	1 (7.1)	1 (7.1)	12 (85.8)	0.428
No	1 (1.5)	8 (12.3)	56 (86.2)	
Socioeconomic status				
Low	1(5.9)	2 (11.8)	14 (82.3)	0.332
Medium	0 (0)	6 (8.1)	68 (91.9)	
High	1 (5.3)	2 (10.6)	16 (84.1)	
Education level				0.833
Basic	1 (3.2)	2 (6.4)	28 (90.4)	
Middle	0 (0)	4 (11.1)	32 (88.9)	
High	1 (2.3)	4 (9.2)	38 (88.5)	
Marital status				0.449
With a partner	1 (30)	4 (8)	45 (90)	
Single	1 (3.3)	5 (16.5)	24 (80.2)	
Glycemic control				0.770
Poor control	1 (1.5)	6 (8.8)	61 (89.7)	
Good Control	1 (2.9)	2 (5.7)	32 (91.4)	

Mean and standard deviation; chi squared test.

## Discussion

Diabetes education is a fundamental component of comprehensive patient care and is essential for achieving metabolic control, as well as reducing or preventing the complications associated with the disease. Information and Communication Technologies (ICTs) are valuable tools, especially in healthcare areas where there is a high demand for medical care.

In the present study, the usability of an educational website on diabetes is reported, with 89% of patients rating the educational tool as highly usable. Nine out of ten patients found it useful, easy to use, and of adequate quality. Authors who have assessed usability in patients with diabetes using an app have consistently rated ease of use and learning highly, while the lowest ratings were for trust and quality of interaction with the app. Additionally, those with a lower educational level tended to rate it more favorably (19). It has been reported that with the implementation of web-based educational programs, patients with lower diabetes education have a higher perception of the risk of complications. In this study, however, no differences in the usability of the educational program were found when comparing it with the level of education or socioeconomic status (24). This could be explained by the fact that the website “Entiendo mi diabetes” included simple, easily understandable information presented interactively to facilitate patient understanding and action in caring for their health. Even though age could be a limiting factor for the use of educational tools with ICTs, no differences in usability were observed.

When considering the dimensions of the usability instrument, more than 85% of the studied patients believed that the educational program met, provided adequate information, and was easy to use. It is important to highlight aspects of quality that the educational content was designed by nutritionists and clinical doctors specializing in diabetes care in primary care clinics. Moreover, it underwent a validation process by clinical experts.

Other authors have assessed usability using the CSUQ program, and this study is one of the few that has demonstrated the level of usability of an educational program, finding a higher rating than what other authors have reported (25, 26).

It was observed that patients perceive an adequate system quality, meeting expectations, and the interface quality proves to be intuitive, generating user satisfaction. These results align with findings from other authors, suggesting that usability improves when a multidisciplinary team is involved in creating educational programs for patients. However, it becomes more valuable when patients are involved in the evaluation process (27).

It is essential to highlight that only 29% of the population had received in-person diabetes education at their clinic. Therefore, continuing the development of web-focused educational materials can be of significant value, enabling patients to receive diabetes education remotely. Additionally, it can serve as a motivating factor for them to manage their condition effectively from the time they receive a diagnosis.

In this regard, healthcare professionals are typically the ones evaluating educational programs. Therefore, it becomes necessary for the system’s quality to meet user expectations, to assess the quality of information and its comprehension, and ensure that the interface is intuitive for the user. Other authors have reported the usability of an app prototype for diabetic foot self-care in end-users, identifying high usability due to the fact that the tool was centered around user needs and requirements (28).

Prior to implementing an educational program using ICTs, evaluation by experts in the medical, nutrition, and diabetes education fields, as well as by software developers for its implementation, is crucial. Finally, the development should undergo assessment by end users. One of the strengths of this study was the design of an instrument to measure usability focused on aspects of diabetes care. The instrument developed had adequate reliability, with a Cronbach’s Alpha value of 0.93. It was found that 98% of participants considered the educational program useful for understanding the disease, for promoting diabetes care and adherence to diet and exercise. To date, there is limited information on patient satisfaction for diabetes management websites in Mexico.

Among the limitations of the present study is the cross-sectional design. Future studies should assess the usability at various stages throughout the duration of an educational program. Additionally, there should be increased participation of women in the study, as they are the ones who most frequently seek medical attention.

It is crucial that healthcare professionals become guides in the use of technologies as new methods of learning. It has been reported that this approach increases patient trust, a better adherence to treatment and motivation in health education can be achieved (29, 30). When the patients become acquainted with an education web site it is necessary and very important the follow-up by healthcare professionals. This contributes to assessing the long-term effectiveness of interventions of ICTs.

On the other hand, in patients with type 2 diabetes in Mexico, poor glycemic control has been reported in 2016 National Health and Nutrition Survey (Ensanut 2016). This survey showed that 68.2% of patients do not achieve metabolic control, furthermore they present a late diagnosis, and high prevalence of overweight and obesity. Then, with this conditions, the sample have a higher risk of developing complications of the disease, such as kidney or cardiovascular disease (5).

There is a high demand for health services for patients with type 2 diabetes in primary care units in Mexico. By the year 2022, it was estimated that the IMSS would care for 4.2 million patients with diabetes. 64% of the care in primary care clinics at the IMSS was for diabetes (31).

According to this high demand for health care, provide diabetes education becomes a complex task. Therefore, digital tools to provide pharmacological treatment and diabetes education at a distance could contribute to improve metabolic control, promove a healthy lifestyle and acquire knowledge of diabetes to avoid complications of the disease. In addition, providing diabetes education through ICTs could reduce the costs of the disease in patients with type 2 diabetes, as well as improve the perception of their quality of life, as previously reported (32).

## Conclusion

The educational diabetes website received a high usability rating from nine out of ten patients. Furthermore, the study revealed that the educational program was deemed useful and easily comprehensible, addressing key aspects to inform and motivate self-care in diabetes. User interaction and satisfaction remained unaffected by factors such as age, education, or socioeconomic status. Assessing usability is crucial for enhancing the end-user experience, with significant implications for adherence and motivation in utilizing digital tools for health education. There is an emphasis on the importance of granting patients access to new knowledge through simple and user-friendly tools, underscoring the need for reinforcement by healthcare professionals.

## Data availability statement

The raw data supporting the conclusions of this article will be made available by the authors, without undue reservation.

## Ethics statement

The studies involving humans were approved by COMITE DE ETICA E INVESTIGACIÓN, INSTITUTO MEXICANO DEL SEGURO SOCIAL. The studies were conducted in accordance with the local legislation and institutional requirements. The participants provided their written informed consent to participate in this study. Written informed consent was obtained from the individual(s) for the publication of any potentially identifiable images or data included in this article.

## Author contributions

GO: Investigation, Validation, Writing – original draft. SV: Investigation, Writing – original draft. CI: Investigation, Writing – original draft, Methodology. AM: Writing – original draft, Conceptualization, Formal analysis. LV: Conceptualization, Formal analysis, Investigation, Methodology, Writing – review & editing.
